# More than Meets the Eye: The Aryl Hydrocarbon Receptor is an Environmental Sensor, Physiological Regulator and a Therapeutic Target in Ocular Disease

**DOI:** 10.3389/ftox.2022.791082

**Published:** 2022-03-03

**Authors:** Christine L. Hammond, Elisa Roztocil, Vardaan Gupta, Steven E. Feldon, Collynn F. Woeller

**Affiliations:** ^1^ Flaum Eye Institute, Rochester, NY, United States; ^2^ Department of Environmental Medicine, School of Medicine and Dentistry, University of Rochester, Rochester, NY, United States

**Keywords:** AHR (aryl hydrocarbon agonist), eye, retina, thyroid eye disease (TED), FICZ, 6-formylindolo (3, 2-b) carbazole, TCDD (2, 8-tetrachlorodibenzo- p-dioxin), cigarette

## Abstract

The aryl hydrocarbon receptor (AHR) is a ligand activated transcription factor originally identified as an environmental sensor of xenobiotic chemicals. However, studies have revealed that the AHR regulates crucial aspects of cell growth and metabolism, development and the immune system. The importance of the AHR and AHR signaling in eye development, toxicology and disease is now being uncovered. The AHR is expressed in many ocular tissues including the retina, choroid, cornea and the orbit. A significant role for the AHR in age-related macular degeneration (AMD), autoimmune uveitis, and other ocular diseases has been identified. Ligands for the AHR are structurally diverse organic molecules from exogenous and endogenous sources. Natural AHR ligands include metabolites of tryptophan and byproducts of the microbiome. Xenobiotic AHR ligands include persistent environmental pollutants such as dioxins, benzo (a) pyrene [B (a) P] and polychlorinated biphenyls (PCBs). Pharmaceutical agents including the proton pump inhibitors, esomeprazole and lansoprazole, and the immunosuppressive drug, leflunomide, activate the AHR. In this review, we highlight the role of the AHR in the eye and discuss how AHR signaling is involved in responding to endogenous and environmental stimuli. We also present the emerging concept that the AHR is a promising therapeutic target for eye disease.

## Introduction

Nearly one billion people in the world have a form of visual impairment (GBD 2019 Blindness and Vision Impairment Collaborators and Vision Loss Expert Group of the Global Burden of Disease Study, 2021). Furthermore, as the population expands and continues to age, this number is expected to increase. A more complete understanding of how the eye and ocular system regulate physiological function and respond to the environment will allow us to better treat or possibly prevent vision impairment. The ocular system is in constant defense against light-induced damage, locally generated reactive oxygen species (ROSs), and toxicants through both direct exposure and the blood stream. Furthermore, the retina is in frequent and direct exposure to light, leading to the formation of photooxidized lipids that are toxic to retinal pigmented epithelial (RPE) cells (Simó et al., 2010). Not surprisingly, since the retina is one of the highest oxygen-consuming tissues in the body, it is also an area of high oxidative stress (Simó et al., 2010).

The ocular surface is directly exposed to environmental pollutants, including cigarette smoke, biomass smoke from wood, coal or other organic material, and ambient particulate matter (Martin et al., 2013; Araj et al., 2020). These environmental components contain polycyclic aromatic hydrocarbons (PAHs) that can serve as ligands of the aryl hydrocarbon receptor (AHR), such as benzo (a) pyrene [B (a) P], dioxins, and hydroquinone (Hu et al., 2013; Julliard et al., 2014; Awji et al., 2015). Cigarette smoking is the main modifiable risk factor for many eye diseases including: age-related macular degeneration (AMD), diabetic retinopathy, glaucoma, thyroid eye disease (TED), and proliferative vitreoretinopathy (PVR) (Eliott et al., 2017; Lee et al., 2020; Molla et al., 2020; Flores et al., 2021; Gupta et al., 2021). Of course, smoke exposure can initiate and contribute to conditions of chronic inflammation and lead to disease (Lee et al., 2012). Additionally, millions of people in the developing world use biomass fuel as a primary means of cooking and heating, resulting in exposure to biomass smoke on a regular basis (West et al., 2013).

The AHR is a key regulator of proliferation, metabolism, inflammation and immune cell signaling. A role for the AHR has been established in hematopoiesis (Bennett et al., 2018), T-cell differentiation (Quintana et al., 2008; Veldhoen et al., 2008), dendritic cell function and regulation of other immune cells (Cella and Colonna, 2015; Esser et al., 2018). Furthermore, ligand-mediated activation of the AHR can mitigate chronic inflammation, including inflammation due to chronic smoke exposure (Baglole et al., 2008; Sarill et al., 2015; Rogers et al., 2017). This dichotomy in the role of the AHR may be due to differences in ligand half-life, binding affinity, timing of activation, cell type, and interactions with other signaling pathways. In this review, we highlight the role of the AHR in the ocular system, as an environmental sensor, normal physiological regulator and potential therapeutic target.

### 1 The Aryl Hydrocarbon Receptor

**FIGURE 1:**
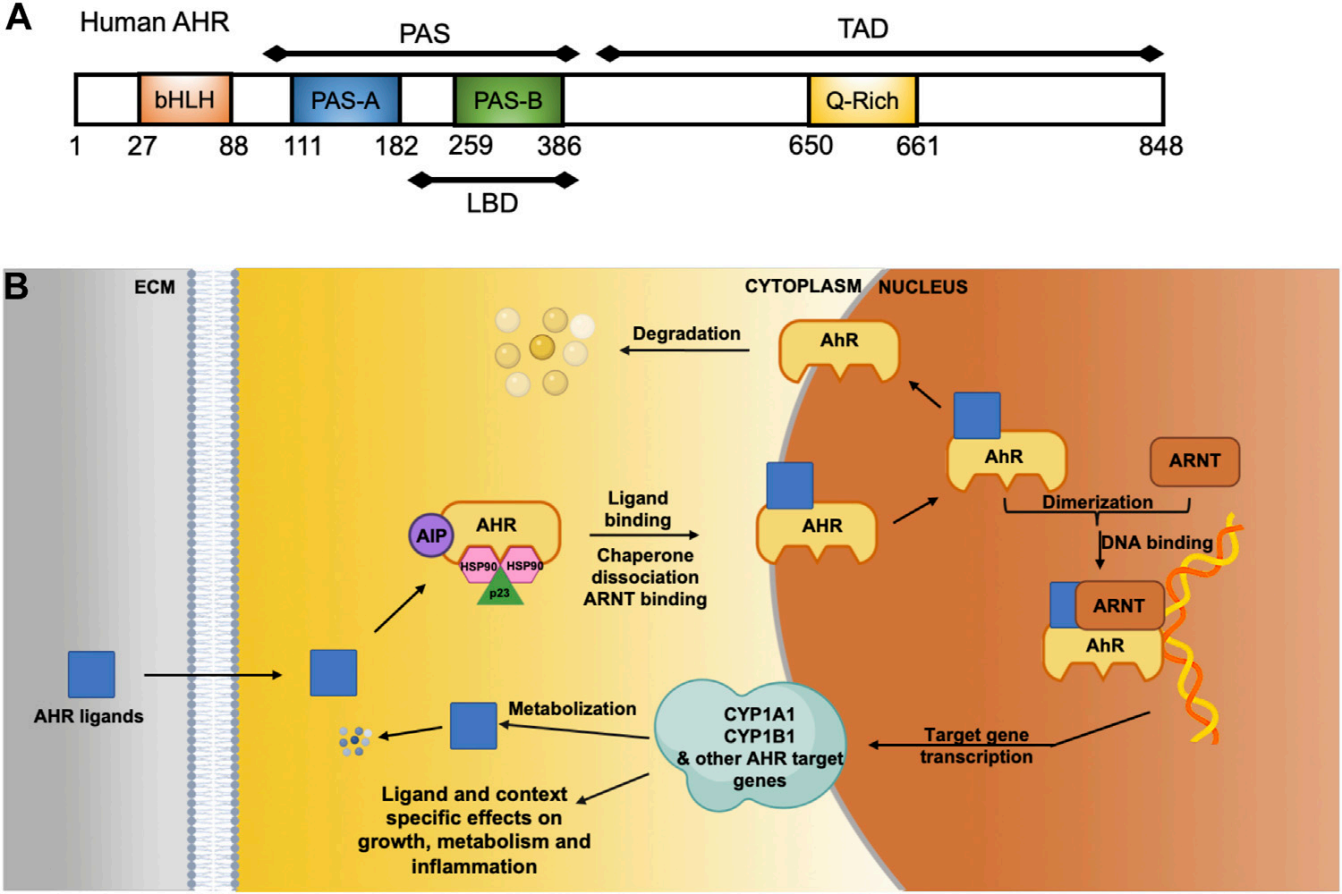
The AHR and its cellular functions as a ligand-activated transcription factor and cytoplasmic modifier. **(A)** Modular diagram of the human AHR protein motifs showing relevant residue numbers (1–848). The AHR contains a basic helix-loop-helix (bHLH) DNA binding domain, a Per-Arnt-Sim (PAS) motif, which includes part of the ligand binding domain (LBD) and at the C-terminal region a transcriptional activation domain that includes a glutamine rich Q-rich) motif. **(B)** Without ligand, AHR is localized to the cytoplasm and bound to chaperone proteins AIP, HSP90 and p23. When and AHR ligands enters the cell, the AHR binds to the AHR nuclear translocator protein (ARNT) in the cytoplasm and is transported into the nucleus where it acts as a transcription factor. AHR-dependent genes include numerous cytochrome P450 enzymes, such as CYP1B1 and CYP1A1, which are involved in metabolism and clearance of xenobiotic polycyclic aromatic hydrocarbons (PAHs).

The AHR is a ligand-activated transcription factor that belongs to the basic helix-loop-helix-PER-ARNT-SIM (bHLH-PAS) family (Figure 1A) (Gasiewicz et al., 2017; Soshilov and Denison, 2008). Distinct AHR ligands bind to the AHR in selective ways and may induce differing AHR dependent responses (including activation of different target genes) (Murray et al., 2010; Wheeler et al., 2014; Esser et al., 2018; Muku et al., 2019). Without bound ligand, the AHR is localized in the cytosol within a protein complex that includes the 90 kDa heat shock protein (HSP90), AHR-interacting protein (AIP), co-chaperone p23, and the protein kinase SRC (Figure 1B) (Carlson and Perdew, 2002; Nukaya et al., 2010). Upon ligand binding, the chaperones are released and the AHR translocates to the nucleus and forms a heterodimer with AHR-nuclear translocator (ARNT). This complex binds to specific DNA binding elements termed dioxin-response elements (DREs), also called xenobiotic response elements (XREs), located in or near target genes. The AHR can activate transcription of hundreds of target genes that are diverse and effect many cellular processes (Denison et al., 2011). The classic AHR target genes include detoxification enzymes, such as cytochrome p450 enzymes (*CYP1A1, CYP1A2* and *CYP1B1*), glutathione-S-transferases (*GST*s) and NAD(P)H-quinone oxidoreductases (*NQ O 1*). The AHR also targets genes involved in lipid metabolism, redox, inflammatory signaling, proliferation, cell communication, angiogenesis, and cell adhesion (Bock and Köhle, 2006; Faust et al., 2013; Bock, 2019). The AHR alters expression of many different genes and signaling pathways independently of XREs. In a non-genomic pathway, AHR activation can regulate Src activity, FAK signaling and Ca2+ flux in the cytoplasm (Puga et al., 2009; Le Vee et al., 2016; Brinchmann et al., 2018; Zhu et al., 2018). Additionally, the AHR functions as an E3-ubiquitin ligase to promote ubiquitin-mediated degradation of other transcription factors including the androgen receptor, the estrogen receptor, retinoic acid receptor, the retinoblastoma protein, and β-catenin (Ohtake et al., 2007; Schneider et al., 2014; Rothhammer and Quintana, 2019). The AHR repressor (AHRR, also an AHR target gene) and the hypoxia-inducible factors 1, 2 and 3 (HIF1α, HIF2α and HIF3α) can compete with AHR binding to ARNT (also called HIF1β), thus preventing AHR driven gene expression (Chan et al., 1999; Sakurai et al., 2017). In a form of negative feedback, the AHR is selectively degraded by the ubiquitin-proteasome system after ligand binding and transcriptional activation (Ma and Baldwin, 2000).

#### 1.1 Aryl Hydrocarbon Receptor Ligands

AHR ligands include structurally diverse persistent environmental pollutants, natural derivatives of tryptophan, and certain pharmaceutical agents (Rothhammer and Quintana, 2019; Shi et al., 2020). The AHR is best known as the receptor that binds 2,3,7,8-tetrachlorodibenzo-*p*-dioxin (TCDD), a persistent organic pollutant that is sometimes simply referred to as dioxin. TCDD is an extremely stable molecule that binds the AHR with high affinity to drive constitutive and untimely activation of the AHR, thus contributing to TCDD-induced toxicological effects (Denison et al., 2011). AHR ligand-dependent toxicity appears to be linked with the metabolic persistence and affinity of binding to the AHR (Denison and Nagy, 2003; Denison et al., 2011). Other persistent pollutant AHR ligands include: 3-methylcholanthrene, B (a) P, β-naphthoflavone, α-naphthoflavone, polychlorinated biphenyls (PCBs) like PCB126, and many other components of cigarette, wood or other biomass generated smoke (Denison and Nagy, 2003; Julliard et al., 2014).

More recently, attention has focused on the discovery of shorter-lived AHR ligands that activate AHR transiently with the potential for beneficial effects and little to no toxicity. Natural AHR ligands, which are often rapidly metabolized after transiently activating the receptor, can be derived from diet, microbiome and metabolic activation (Rothhammer and Quintana, 2019). Dietary components can be metabolized or modified by the microbiome into AHR agonists (Rothhammer and Quintana, 2019; Shi et al., 2020). AHR ligands derived from the diet include indoles, such as indole-3-carbinol and tryptophan metabolites, like kynurenine and kynurenic acid (Hubbard et al., 2015; Rothhammer and Quintana, 2019). Flavonoids such as quercetin and curcumin are also implicated as AHR activators (Ciolino et al., 1999; Mohammadi-Bardbori et al., 2012). Another identified ligand is 6-formylindolo [3,2-b]carbazole (FICZ), which can be formed from UVB-mediated oxidation of tryptophan in the skin or generated from indole-3-aldehyde (Rannug and Rannug, 2018; Bock, 2019). These shorter-lived AHR ligands possess promising anti-inflammatory and pro-resolution properties that maintain homeostasis and may be beneficial to treat disease (Rannug and Rannug, 2018; Rothhammer and Quintana, 2019).

Additional natural and synthetic AHR agonists are being identified and developed for disease specific applications. Tapinarof, a natural AHR ligand produced by some bacteria is being evaluated to treat skin inflammation (van den Bogaard and Perdew, 2021; Smith et al., 2017). Currently used pharmaceutical compounds that activate the AHR have been identified. These include: the proton pump inhibitors, esomeprazole (Nexium) and lansoprazole (Prevacid), the immunosuppressive drug, leflunomide, and flutamide, an antiandrogen (Hu et al., 2007; Jin et al., 2012; Novotna et al., 2014; Yamashita et al., 2014). Additionally, novel and selective AHR modifier compounds, like VAF347 [(4-(3-Chloro-phenyl)-pyrimidin-2-yl)-(4-trifluoromethyl-phenyl)-amine], and the indole containing ligand 2AI, are being created to elicit a desired level of AHR activation for treating inflammatory diseases, including those of the retina (Gutierrez et al., 2016; Zapadka et al., 2021).

#### 1.2 Aryl Hydrocarbon Receptor Signaling Pathway Crosstalk

The AHR signaling pathway involves crosstalk with several major cell signaling pathways mediating cell proliferation, metabolism, immune regulation, inflammation and tissue remodeling. Other reviews have covered many of these pathways, and we only highlight three pathways in the ocular system (Puga et al., 2009; Schneider et al., 2014; Jaeger and Tischkau, 2016; Button et al., 2017; Rothhammer and Quintana, 2019). The transforming growth factor beta (TGFβ), wingless-related MMTV integration site (wnt) and hypoxia inducible factor (HIF) pathways crosstalk with AHR (Mathew et al., 2009; Puga et al., 2009; Faust et al., 2013). These pathways play fundamental roles in eye development and disease (Kurihara et al., 2012; Bennis et al., 2017; Jeon et al., 2018). The TGFβ pathway, which is driven by the extracellular cytokines TGFβ-1, 2 and 3, governs cell growth, differentiation, wound healing, and scarring (fibrosis). Ocular diseases that show scar formation, including corneal scarring, PVR and TED, exhibit robust TGFβ activity (Hindman et al., 2010; Jeon et al., 2014; Woeller et al., 2016; Heffer et al., 2019). AHR decreases TGFβ signaling; whereas, a reduction in the AHR enhances TGFβ signaling (Woeller et al., 2016). The AHR-TGFβ crosstalk is complex because in some cell types, TGFβ signaling alters AHR expression and activity (Gomez-Duran et al., 2009).

The wnt signaling pathway is important for development, proliferation, differentiation, and migration. Dysregulation of wnt signaling has been reported in eye diseases including TED and PVR (Ezra et al., 2012; Chen et al., 2015; Woeller et al., 2016). In general, AHR activation appears to downregulate wnt signaling, however in some cell types and species-specific events, wnt signaling may be induced by AHR activation (Schneider et al., 2014).

The HIF pathway, mediated by three HIF transcription factors, HIF-1α, 2α, 3α, is important for oxygen homeostasis, angiogenesis, glucose metabolism, cell survival, and activation of the immune system (Ratcliffe, 2007; Loboda et al., 2010; Vorrink and Domann, 2014; Button et al., 2017). In normal oxygen environments, HIFs are targeted for proteasomal degradation; however, when oxygen is low, they accumulate and translocate to the nucleus to activate transcription of target genes. Like AHR, HIFs dimerize with ARNT and in doing so, HIFs potentially compete with the AHR. Thus, AHR-mediated transcription may decrease with elevated HIF activity (Button et al., 2017). HIF-2α was shown to mediate fibrotic signaling in orbital fibroblasts from TED patients (Hikage et al., 2019). Furthermore, chronic cigarette smoking, which is a major risk factor for ocular diseases including AMD, TED and PVR (Gupta et al., 2021), leads to increases in systemic hypoxia (Fricker et al., 2018), likely increasing HIF activation at the expense of the AHR pathway.

## 2 Aryl Hydrocarbon Receptor Expression in the Eye

**FIGURE 2:**
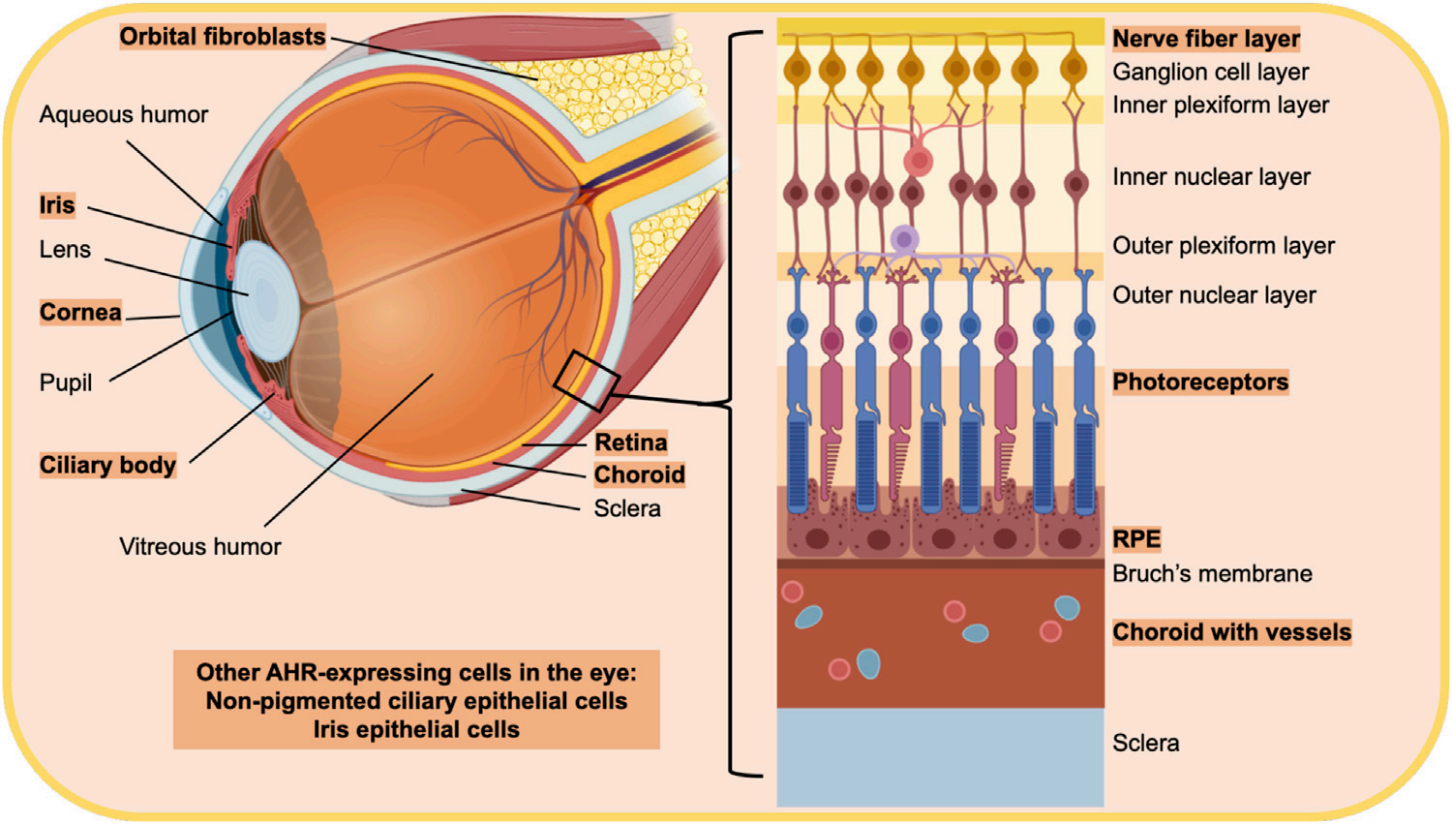
Diagram of the eye highlighting regions and cells that have been shown to express the AHR. The text in orange highlight indicates cells and tissue that express the AHR. The eye is a unique sensory organ, composed of three main layers. The outer layer is composed of the sclera and cornea, which are directly exposed to the environment on the surface of the eye. The middle layer is made up of the ciliary body, iris and choroid while the inner most layer is the retina. Other parts of the ocular system include the fluid filled vitreous in between the retina, and ciliary body and the lens. Orbital tissue behind the eye provides structural support and protects the optic nerve. Retinal pigment epithelial cells (RPE), choroid, the ciliary body and non-pigmented ciliary epithelial cells, orbital fibroblasts and the cornea have all been shown to express the AHR.

AHR-dependent genes, including CYP1A1 and CYP1B1, mediate retinoic acid metabolism (Chen et al., 2000; Chambers et al., 2007). Retinoic acid and its metabolites are essential for proper eye development and function. Therefore, AHR expression and signaling in the eye is likely essential for normal ocular system function. The AHR in general is expressed ubiquitously, in all mammalian cells and tissues, however, expression levels vary widely. This is also true within the eye (Okey, 2007; Choudhary and Malek, 2020) (Figure 2).

AHR transcript levels increase in postnatal development of mice in both retinal cells and in rod photoreceptors (Kim et al., 2014). AHR expression increases in prenatal development to a peak at a young age, and then AHR activity declines with increasing age (Okey, 2007; Hu et al., 2013). Eye structures with high AHR expression or activity include the retina and choroid, especially RPE cells (Shichi and Nebert, 1982; Zhao and Shichi, 1995; Carvalho and Tillitt, 2004; Jiang et al., 2010; Dwyer et al., 2011; Hu et al., 2013; Choudhary et al., 2015). The iris epithelium also has high AHR expression (Bennis et al., 2017). Additional areas of pronounced AHR expression are in the ciliary body and non-pigmented ciliary epithelial cells (Zhao and Shichi, 1995; Volotinen et al., 2009). Other structures related to the eye that have detectable AHR expression include the cornea and orbital fibroblasts (Shichi and Nebert, 1982; Zhao and Shichi, 1995; Woeller et al., 2016). In the lens, AHR expression is below detectable levels (Shichi and Nebert, 1982; Zhao and Shichi, 1995). However, the AHR appears to regulate the αβ-crystallin gene, which is highly expressed in the mouse lens and is downregulated in AHR-knock-out mice, suggesting the presence of AHR in the lens.

Phenotypically, AHR knockout mice do not exhibit traits of low vision that interfere with successful maturation, however, these mice do show abnormal vascular structures in the eye (Lahvis et al., 2000). AHR deficient mice present nystagmus (rapid involuntary eye movements) (Chevallier et al., 2013) and show increased RPE autofluorescence, sub-RPE deposits, and RPE degeneration in older mice (Hu et al., 2013). Interestingly, RPE degradation in AHR knockout mice is similar to the atrophic lesions observed in humans with AMD (Kim et al., 2014). Further, retinal thinning, photoreceptor loss (Zhou et al., 2018) and choroidal atrophy are observed in aged AHR-knockout mice (Hu et al., 2013). In Drosophila, the AHR ortholog spineless is required for the development of color vision (Wernet et al., 2006).

Other studies have shown that AHR expression or activity is increased in response to light. An *in vitro* study using human RPE cells showed that blue light increased AHR activity, as demonstrated by increases in *AHR, CYP1A1* and *NQ O 1* (Gutierrez et al., 2016). Both TCDD and 2AI protect RPE cells from toxicity due to 4-hydroxynonenal, the lipid peroxidation product produced after light exposure (Gutierrez et al., 2016). The AHR is expressed in mature mouse photoreceptors (Kim et al., 2014; Gutierrez et al., 2016), and after light exposure, AHR is expressed in the nuclei (suggesting ligand binding and nuclear translocation) of photoreceptor cells, inner retinal cells, and RPE cells of mice (Gutierrez et al., 2016). Light exposure in mice also increases CYP1A1 in the retina and RPE cells (Gutierrez et al., 2016). Since the AHR ligand FICZ can be generated by photooxidation of tryptophan metabolites (Rannug and Rannug, 2018), it may be that light contributes to natural FICZ production in the eye. Indole-3-carbinol, a naturally derived AHR agonist, prevents light-induced retinal damage in C57BL/6 mice and also attenuates light-induced inflammatory markers such as IL-1β and IL-6 (Khan and Langmann, 2020). The indole containing the AHR ligand 2AI also protects C57BL/6 mouse retinas from light-induced damage (Gutierrez et al., 2016).

## 3 Environmental Pollutants That Disrupt Endogenous Aryl Hydrocarbon Receptor Functions in the Eye

AHR is a critical environmental sensor that robustly responds to environmental pollutants (Carlson and Perdew, 2002; Ko et al., 2014; Esser and Rannug, 2015; Kim et al., 2017; Nebert, 2017; Rothhammer and Quintana, 2019). Here, we focus on the ability of AHR to respond to pollutants in the eye. TCDD, B (a) P, β-naphthoflavone, α-naphthoflavone, and hydroquinone all activate the AHR as measured by the upregulation of AHR responsive genes in human RPE cells (Hu et al., 2013) or RF/6A choroid endothelial cells (Choudhary et al., 2015). Animal model studies have uncovered developmental toxicity to the eye due to TCDD exposure and subsequent AHR activation (Mattingly et al., 2001; Blankenship et al., 2003; Carvalho and Tillitt, 2004; Miettinen et al., 2004; Takeuchi et al., 2009; Liu and Piatigorsky, 2011; Garcia et al., 2017; Choudhary and Malek, 2020). For example, TCDD exposure leads to a dose-dependent decrease in retinal ganglion cells (RGCs) in the swim-up developmental stage of trout, which decreases vision (Carvalho and Tillitt, 2004). RGCs are developmentally the first retinal neurons present in fish and are responsible for the synaptic link between the retina and the dorsal midbrain (Carvalho and Tillitt, 2004). TCDD exposure also leads to an increase in apoptotic cells in the eye of the Atlantic kill fish, *Fundulus heteroclitus* (Toomey et al., 2001). In zebrafish, treating embryos with PCB126 changes expression of several vision related genes, including CYP1B1 and opsin genes (Meyer-Alert et al., 2021). Organic constituents (mostly PAHs and quinones) isolated from ambient airborne particulate matter activated AHR and disrupted gene expression in the eyes of zebrafish embryos (Mesquita et al., 2015). While these studies show a clear role for pollutant derived AHR ligands to disrupt endogenous AHR signaling in the eye, the role of the pollutants in driving human ocular disease requires further study.

### 3.1 Ocular Diseases and the Potential Role of Aryl Hydrocarbon Receptor

#### 3.1.1 Uveitis

Uveitis is characterized by swelling and inflammation of the choroid, ciliary body, and iris, all of which comprise the uvea. There are over 10 proposed self-antigens responsible for inducing uveitis in humans (Caspi, 2010). Some uveitis patients produce autoantibodies to the interphotoreceptor retinoid-binding protein (IRBP) (Zhang et al., 2010). IRBP is a large glycoprotein synthesized by photoreceptors and released into the subretinal space. IRBP is important for solubilizing retinol and retinal, fatty acid transport, and for the maintenance of photoreceptors (Simó et al., 2010). Experimental autoimmune uveitis (EAU) is an animal disease model for human uveitis. EAU in mice is often induced by immunization with IRBP (Caspi, 2010). In this disease, immune cells including macrophages and T helper 17 lymphocytes (T_H_17 cells) cross the blood-retinal barrier (BRB) and damage the retina. Activated macrophages and retinal microglia release TNFα and iNOS and lead to inflammation and apoptosis of retinal cells causing vision loss.

In AHR knockout mice, the development of EAU is more severe than in wild-type mice, with higher levels of macrophage and microglial recruitment (Huang et al., 2018). Levels of pro-inflammatory cytokines, including TNFα, IL-6 and IL-1β, are also higher in AHR-knockout animals.

#### 3.1.2 Infantile Nystagmus Syndrome

Nystagmus is an involuntary movement of the eye that disrupts the gaze fixation on an object. It is characterized as a drifting of the eye away from an intended object and a return to the intended gaze direction, which can be quick or slow. In INS, these involuntary movements occur randomly and reduce the ability to maintain the gaze on particular objects and thus decreases vision. Three children experiencing INS from the same family were found to have a loss of function mutation of the *AHR* gene. The resulting AHR protein is truncated and missing a portion of the C-terminus (Mayer et al., 2019; Borovok et al., 2020). AHR protein expression and function in these patients is decreased to at least half of the level of controls (Borovok et al., 2020). As previously mentioned, nystagmus is also observed in AHR knockout mice (Chevallier et al., 2013). Further study of these AHR knockout mice revealed that the mice with nystagmus also have deficiencies in the optic nerve myelin sheath (Juricek et al., 2017). The increase in optic nerve inflammation leads to an attenuation of myelin-associated glycoproteins and elevated IL-1β levels, RANTES (regulated on activated-normal T-cell expressed and secreted), TNFα, and monocyte chemoattractant proteins (MCP) 1–3 (Juricek et al., 2017).

#### 3.1.3 Glaucoma

Glaucoma is related to an increase in intraocular pressure that damages the optic nerve and leads to vision loss and blindness. *CYP1B1,* an AHR-inducible gene, is associated with various forms of glaucoma, including the two major types: primary open-angle glaucoma and primary congenital glaucoma (Vasiliou and Gonzalez, 2008). In a study using the Adverse Outcome Pathway framework, the AHR is linked with glaucoma, including changes in the gene for *CYP1B1* (Oki and Edwards, 2016). At least 82 mutations have been identified in *CYP1B1* and are associated with various forms of glaucoma (Vasiliou and Gonzalez, 2008). Low or absent CYP1B1 activity is associated with abnormal development of the trabecular meshwork (Vasiliou and Gonzalez, 2008). The trabecular meshwork affects intraocular pressure by controlling the outward drainage of the aqueous humor from the anterior chamber of the eye (Abu-Hassan et al., 2014).

TCDD can also induce CYP1B1 expression in human nonpigmented ciliary epithelial cells (Volotinen et al., 2009). These epithelial cells are what make up the ciliary body, the principal function of which is the production of aqueous humor in the eye (Abu-Hassan et al., 2014). CYP1B1 is involved in the metabolism of steroids, retinol and retinal, arachidonate, and melatonin. Therefore, CYP1B1 expression, which is increased by AHR, alters production of critical metabolites and metabolic pathways that may lead to the development and/or progression of glaucoma (Vasiliou and Gonzalez, 2008).

#### 3.1.4 Retinitis Pigmentosa

RP is characterized by night blindness and a progressive loss of peripheral vision that ultimately culminates in complete blindness. RPE atrophy and accumulation of intraretinal pigment deposits contribute to the decline in vision (Zhou et al., 2018). RP is a group of hereditary degenerative diseases of the retina and thought to be associated with mutations in over 70 genes that are expressed in photoreceptors and RPE. However, diseases associated with these genes are only found in approximately half of patients, leaving many causes of disease unknown (Zhou et al., 2018). In one family, two children with RP were found to have homozygous alleles of the same loss of function mutation in the *AHR* gene while unaffected family members were either heterozygous or non-carriers of the mutant allele (Zhou et al., 2018). Conditional, transgenic mice were developed to knockout AHR expression exclusively in the retina. These mice revealed that the loss of AHR in the retina contributes to the development of features of RP. These characteristics have been identified as retinal thinning and loss of photoreceptors in aged mice (∼18 months old) (Zhou et al., 2018).

#### 3.1.5 Age-Related Macular Degeneration

AMD is the leading cause of vision loss in older adults in the US. It is a progressive loss of central vision that culminates in irreversible blindness. Cigarette smoking is one of the largest modifiable risk factors for AMD (Takeuchi et al., 2009). The aging of the eye leads to the buildup of small yellow or white deposits (Drusen) where the RPE, Bruch’s membrane, and neurosensory retina interact. The buildup of drusen and chronic inflammation, part of the pathophysiology of AMD, lead to the destruction of RPE and photoreceptors. Two types of AMD exist, the wet (or exudative) and dry form. In the dry form, there is thinning and degradation of capillaries in the choroid, expanding atrophy of the outer retina, and permanent damage to the photoreceptors mostly resulting in a slow and progressive loss of vision that may take years. Although not as prevalent as dry AMD, wet AMD is more severe and vision loss is more rapid. In wet AMD, the presence of pro-inflammatory cytokines, and in particular vascular endothelial growth factor (VEGF), leads to an increase in angiogenesis and vascular permeability. Newly created blood vessels leak fluid within the retina, damaging photoreceptors and leading to fibrosis and atrophy of the macula. The only effective treatment for stalling progression of the disease is for the wet form, which includes the injection of VEGF inhibitors into the vitreous (Flores et al., 2021).

Expression of the AHR decreases during aging (Okey, 2007; Hu et al., 2013; Kim et al., 2014) and, therefore, the effects of depleting the AHR were studied in RPE and choroidal cell lines. Gene expression of the pro-angiogenic vascular endothelial growth factor A (*VEGFA*) and pro-inflammatory chemokine (C-C motif) ligand 2 (*CCL2*) were increased by depletion of AHR using AHR siRNA in the human RPE cell line, ARPE19 (Choudhary et al., 2015). AHR depletion elevates collagen IV production and secretion in ARPE19 and RF/6A choroid cells (isolated from Rhesus macaque) (Choudhary et al., 2015). AHR depletion in RF/6A cells also increases gene expression of the macrophage chemotactic factor, secreted phosphoprotein 1 (*SPP1*) and *TGFβ* while decreasing expression of anti-angiogenic factor *SERPINF1* (Choudhary et al., 2015). Mice exposed to TCDD showed increased levels of VEGFA and choroidal vascularization, both being hallmarks of AMD (Takeuchi et al., 2009). These results suggest that the loss of AHR expression or TCDD (and/or other toxicants in cigarette smoke)-induced activation of AHR contributes to angiogenesis, inflammation, and alterations in the extracellular matrix, all of which are observed in wet AMD. Introduction of choroidal neovascular lesions, to propagate a pathophysiology similar to wet AMD in AHR knockout mice, lead to larger lesions, increased microglial cell recruitment, and elevated collagen IV deposition compared to wild-type mice (Choudhary et al., 2015).

AHR knockout mice develop a dry AMD-like pathology as they age (Hu et al., 2013; Kim et al., 2014). These changes at 11–12 months of age include: loss of RPE tight junctions (indicating a breakdown of the BRB), increased autofluorescence within the retina, and increased RPE and choroidal atrophy (Hu et al., 2013; Kim et al., 2014). In even older AHR knockout animals (16 months), deposits and debris are observed in the Bruch’s membrane, which is thicker due to an increase in the inner collagenous layer and/or the elastic layer (Hu et al., 2013). This is consistent with reported changes in human AMD histological sections.

## 4 Therapeutic Potential of Aryl Hydrocarbon Receptor Ligands

**FIGURE 3:**
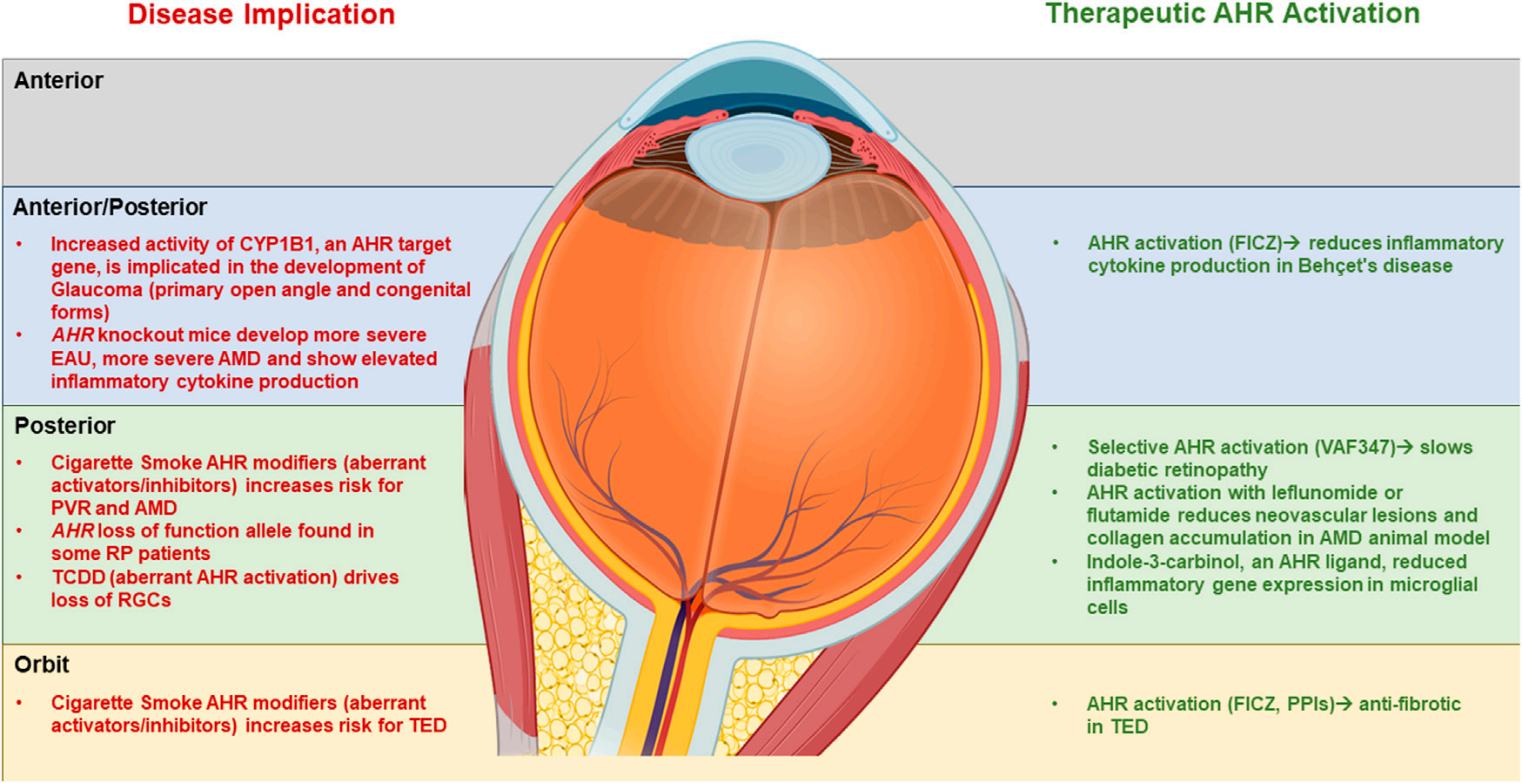
Summary of the role of AHR in eye disease and the therapeutic potential of AHR activation AHR. A summary of the role of AHR in development of eye disease (left side) as compartmentalized into Anterior, posterior and orbital sections of the ocular system. On the right side is a summary of the potential use of AHR ligands in treating ocular disease.

While the AHR may have a role in regulating eye development, sensing environmental pollutants and ocular disease, several studies have revealed that activating the AHR using AHR ligands may be a novel mechanism to treat ocular diseases (Figure 3). For example, in the EAU mouse model, activating AHR with TCDD 1 day before IRBP immunization in prevents EAU from developing (Zhang et al., 2010). TCDD exposure decreased TNFα, IL-6, and IL-1β activation by EAU and inhibited NF-κB and STAT pathways (Huang et al., 2018). The AHR ligand ITE also inhibits EAU development in mice (Nugent et al., 2013).

AHR activation may also be useful in treating Behçet’s disease. Behçet’s disease is a rare autoinflammatory disease that causes blood vessel inflammation throughout the body, including in the eye. This inflammation can lead to chronic uveitis, mouth ulcers, and skin lesions (Chi et al., 2008). When treated with lipopolysaccharide (LPS), dendritic cells isolated from Behçet’s disease patients produce more IL-1β, IL-6, IL-23, and TNFα than dendritic cells from control groups (Wang et al., 2014a). Activation of the AHR by FICZ or ITE inhibits dendritic cell differentiation and function, which leads to an inhibition of T_H_1 and T_H_17 cell responses (Wang et al., 2014a). These AHR ligands inhibit cytokine expression from dendritic cells including TNFα, IL-1β, IL-6, and IL-23 (Wang et al., 2014a). Additionally, gene expression of the AHR is decreased in PBMCs isolated from Behçet’s disease patients compared with control (Wang et al., 2014b).

Diabetic retinopathy is the leading cause of vision loss in the working-age population of developed countries (Simó et al., 2010; Semeraro et al., 2015; Zapadka et al., 2021). In type one diabetes, proliferative diabetic retinopathy is common and includes hypoxia-induced neovascularization (Simó et al., 2010). In type two diabetes, the primary cause of vision loss is diabetic macular edema, which is mainly due to the breakdown of the BRB and resulting vascular leakage. In diabetic retinopathy, antioxidants such as glutathione, superoxide dismutase, and ascorbic acid are diminished leaving the retina more susceptible to damage cause by oxidative stress (Simó et al., 2010). Diabetes also leads to tissue hypoxia and chronic low-grade inflammation in the retina (Semeraro et al., 2015). The streptozotocin-induced diabetic mouse model demonstrates retinal degeneration and capillary destruction due to inflammation and elevated levels of IL-17 (Lindstrom et al., 2019; Sigurdardottir et al., 2019). VAF347, a selective AHR agonist, was discovered during a screen for compounds that block IgE production by B lymphocytes (Ettmayer et al., 2006). VAF347 exhibits anti-inflammatory effects by blocking dendritic cell function (Ettmayer et al., 2006; Lawrence et al., 2008). In streptozotocin-treated mice, VAF347 treatment attenuated inflammation and early-stage diabetic retinopathy changes (Zapadka et al., 2021). These data suggest that AHR activation has the potential to prevent and treat early stages of diabetic retinopathy and other diseases that involve elevated IL-17 levels.

Other studies have suggested that activation of AHR could also be therapeutically beneficial in AMD. Two AHR active pharmaceuticals (leflunomide and flutamide) inhibit VEGF-induced RF/6A cell migration and tube formation. Leflunomide and flutamide also improve the severity of choroidal neovascular lesions and decrease the amount of collagen IV that accumulates in the mouse model of AMD (Choudhary et al., 2018). In this study, injections of the drugs started 2 days prior to laser photocoagulation and continued daily for 21 days. Leflunomide and flutamide reduced the inflammatory cytokines IL-12, IL-13, IL-17A, KC, leptin, and MIP-2 in this animal model (Choudhary et al., 2018). The drugs also activated CYP1A1 and CYP1A2 in the RPE/choroid tissue complex of these mice, demonstrating AHR activation. Leflunomide and flutamide are not effective in AHR knockout animals demonstrating that AHR is required for their beneficial effect. Another study showed anti-inflammatory properties of AHR activation in microglial cells. Indole-3-carbinol, an AHR ligand, reduced LPS-mediated pro-inflammatory gene expression in BV-2 microglial cells (Khan and Langmann, 2020).

Thyroid eye disease (TED) is an autoimmune disease that can develop in patients with autoimmune thyroid diseases (Gupta et al., 2021). TED is thought to arise due to the presence of autoantibodies in the orbital tissue behind the eye which leads to remodeling. This remodeling includes the accumulation of fat tissue, muscle tissue, extracellular matrix, scar tissue and edema. These changes then lead to the signs and symptoms of TED including: exophthalmos (eye protrusion), eye lid retraction, dry eye and corneal scarring, diplopia (double vison), and optic nerve damage. The remodeling of orbital tissue in TED is thought to be initiated by an inflammatory response and activation of orbital fibroblasts that express thyroid hormone-stimulating receptor (TSHR), which is considered the primary autoantigen in TED (Gupta et al., 2021).

The AHR is expressed in human orbital fibroblasts (Woeller et al., 2016) and the AHR ligands FICZ, ITE, and the proton pump inhibitors esomeprazole and lansoprazole (which activate the AHR) inhibit TGFβ-mediated myofibroblast differentiation (Woeller et al., 2016; Hammond et al., 2019). FICZ, ITE, esomeprazole, and lansoprazole attenuate TGFβ-induced alpha smooth muscle actin (αSMA) expression and also prevent collagen production and cell migration (Woeller et al., 2016; Hammond et al., 2019). The actions of FICZ, ITE, esomeprazole, and lansoprazole were confirmed to be AHR-dependent as determined through *AHR* knockdown experiments (Woeller et al., 2016; Hammond et al., 2019). TED orbital fibroblasts treated with FICZ also decreased TGFβ-induced collagen production by upregulating matrix metalloproteinase-1 (MMP1), a type 1 collagen degrading enzyme (Woeller et al., 2016; Roztocil et al., 2020). These data suggest that activation of the AHR through natural ligands or pharmaceuticals may be a novel way to mitigate scar formation in TED.

## 5 Conclusion

In summary, the AHR is involved in many different aspects of ocular physiology and has a key role in eye development and regulating inflammatory signaling. Disruption of endogenous AHR signaling through abnormal signaling mediated by pollutant derived ligands or even mutation in the receptor itself may be driving mechanism(s) behind many instances of eye pathophysiology. A thorough understanding of the role that the AHR plays in the ocular system could provide novel avenues for targeting eye disease. AHR ligands are diverse and can have a multitude of effects depending upon the characteristics of the ligand. Understanding these differences by choosing natural or short-lived synthetic ligands to promote the desired effect, or by the creation of novel AHR ligands that selectively induce beneficial effects may provide new and exciting therapies for the treatment of eye disease.
